# Israel should build capacity in implementation science

**DOI:** 10.1186/s13584-025-00669-5

**Published:** 2025-04-02

**Authors:** Adam J. Rose, Sivan Spitzer, Moriah E. Ellen

**Affiliations:** 1 Hebrew University School of Public Health, Jerusalem, Israel; 2 Azrieli Faculty of Medicine, Bar Ilan University, Tsfat, Israel; 3 Department of Health Policy and Management, Guilford Glazer Faculty of Business and Management, Faculty of Health Sciences, Ben-Gurion University of the Negev, Beer Sheva, Israel; 4 Israel Implementation Science and Policy Engagement Centre, Ben-Gurion University of the Negev, Beer Sheva, Israel; 5 Institute of Health Policy Management and Evaluation, Dalla Lana School of Public Health, University of Toronto, Toronto, Canada; 6 Braun School of Public Health, Hebrew University, Ein Kerem Campus, Jerusalem, Israel

**Keywords:** Implementation science, Israel, Health policy, Health services administration

## Abstract

**Background:**

Implementation Science (IS) is a scientific discipline that has been in existence for approximately thirty years. The goal of this discipline is to develop and refine rigorous approaches to producing change in the health system, and thereby to shrink the quality gap between best practice and current practice more quickly and more completely than could occur through naturalistic change alone.

**Main body:**

In this perspective, we review two prominent examples of health systems that invested in building capacity for IS– the Veterans Affairs Health System and Intermountain Healthcare in the United States– and how this investment has catalyzed system-level improvements over time. We make the case that Israel should similarly invest in building IS capacity.

**Conclusion:**

Investing in building IS capacity does not produce quick results, and is not easy. Nevertheless, a plan to build IS capacity should be an important ingredient in our plan to improve Israel’s health system over time.

## Background

### How does change occur in health systems?

Rogers’ theory of diffusion of innovations is the preeminent theory of how new practices come to be widely adopted within systems. Developed over decades, and concretized in Rogers’ book of 1962 [1], the theory states that innovations spread more or less completely or quickly through a social system based on characteristics of the innovation, the individuals, and the organization. There are examples of features that are known to lead to speedy adoption of innovations. For example, an innovation that produces a very large advantage compared to what came before, such as the invention of penicillin, will tend to be adopted comparatively quickly and completely compared to those that produces only a slight benefit. Similarly, a system with strong interconnection among its parts, and leaders who are relatively supportive of change, will tend to adopt innovations more rapidly.

Of note, Rogers’ theory does not always describe the uptake a technological advance such as the development of a new drug, device, or imaging test. In many ways, the development over time of the very idea of evidence-based practice demands that the healthcare system must deliver care in a way that minimizes unnecessary variation in care and that maximizes adherence to evidence about which practices will produce the best results for patients [2]. While evidence-based practice may not seem like a new idea, it can be argued that there still exists within the health system considerable resistance and other barriers to its full adoption, and that Rogers’ theory is still a fitting one to understand the barriers and facilitators of its more complete adoption.

Rogers’ theory can be said to describe *naturalistic* change in a system– that is, adopting a new practice, when nobody is particularly interested in pushing for change in a directive way. However, there can be issues with waiting for naturalistic change to occur. A body of work has shown that in healthcare, it takes on average seventeen years for evidence-based innovations to be fully adopted into practice [3], and some improvements may take longer. If a new way of doing things is truly better, we do not have the luxury of being patient while this naturalistic change plays out. The desire to close quality gaps more quickly led to the development of a new scientific discipline: Implementation Science (IS).

## Main text

### What is implementation science?

A prominent official definition of IS, provided by Eccles and Mittman, is as follows: “Implementation research is the scientific study of methods to promote the systematic uptake of research findings and other evidence-based practices into routine practice, and, hence, to improve the quality and effectiveness of health services. It includes the study of influences on healthcare professional and organizational behavior.” [4] IS is extremely multidisciplinary, with contributions from fields as diverse as medicine, nursing, anthropology, psychology, sociology, and human factors engineering. While we will explain the basics of IS as briefly as possible, there are also well-written review articles [5] to introduce it to the uninitiated in greater detail than space permits us.

IS examines how to effectively translate research findings into real-world practice. While traditional clinical research determines whether interventions improve health outcomes, IS focuses on understanding and optimizing how these proven interventions can be successfully adopted, sustained, and scaled in actual healthcare settings. This field recognizes that bringing evidence-based practices into routine care requires navigating multiple levels of complexity - from individual clinician behavior and organizational systems to broader policy environments. Implementation scientists develop and evaluate strategies to overcome barriers to adoption, studying not just whether an intervention works, but how to best support its consistent, high-quality delivery across diverse healthcare contexts. By bridging the gap between what we know works and how to make it work in practice, IS accelerates the impact of healthcare innovations [5, 6]. 

IS aims to develop approaches to producing health system change that will work in many settings– even in different countries or in countries of dissimilar levels of economic development. This is often referred to in IS as trying to build “generalizable approaches” to promoting innovations that are able to work relatively well in a variety of settings. An example of such an approach to producing health system change, and a mainstay of IS projects, is that of audit and feedback. With sufficient data, providers or groups of providers can be provided with detailed and timely feedback about how their practice compares to others and how it is changing over time. This feedback can help promote desired changes in practice over time [7]. In fact, one review identified 73 distinct strategies that have been used to promote health system change as part of IS initiatives [8]. These strategies are not used in isolation, but often 3–5 of them are used in concert– chosen to address different perceived barriers to the implementation effort.

There is no reason why IS cannot be used in fields other than healthcare. There is also clear applicability to public health interventions located outside the healthcare system, but the impact of IS has reached even farther afield. Popular areas for the use of such approaches to producing system change and promoting evidence-based practice include the correctional system [9, 10], the educational system [11, 12], and many others. Indeed, IS practitioners in education may have much to learn from those who work in healthcare, and vice-versa.

### Comparing implementation science with adjacent fields of inquiry

One way to better understand the field of IS is to contrast it with neighboring fields of inquiry. IS differs from quality improvement (QI) in that QI tends to focus on simpler issues and tends to pursue more ad-hoc solutions to local problems, which may not be generalizable to other systems and may not aim to be. While QI projects often do include impact assessments, they may be in the form of plan-do-study-act (PDSA) cycles, and may be limited in scope and ambition. Due to the lack of generalizability and often-rudimentary impact assessments, it can be hard to publish about QI efforts, although some sophisticated QI projects do lead to publications, and others do not have a goal of being publishable.

In contrast, IS aims to develop and refine approaches to producing change that may be useful to achieve many goals, whether in departments of psychiatry, in cancer screening, or in reducing overlapping prescriptions. In addition, IS is often characterized by more ambitious approaches to impact assessment, including mixed methods and state-of-the-art study designs to support causal inference.

It is important to point out, however, that while IS is often contrasted with QI, the two fields borrow approaches from each other and in fact some degree of convergence between them has been noted. The most ambitious QI projects can come to resemble IS, and may blur the boundary between them. Approaches such as audit and feedback, discussed above, have their origin in QI, but have become a mainstay of IS as well. Many prominent thinkers have recently pointed out that while QI and IS are not the same, they have much to learn from each other in pursuit of the broader goal of improving the healthcare system [13, 14]. 

An additional field of inquiry that is closely related to IS is dissemination science. They are complementary domains that address different aspects of the evidence-to-practice pipeline. Dissemination science focuses on the targeted distribution of evidence-based information to specific audiences, studying how research findings are communicated and spread across healthcare systems [15]. IS, however, moves beyond information sharing to examine the systematic uptake of evidence-based practices into routine care, including the study of influences on professional and organizational behavior change [5, 15]. 

The issue of generalizability is actually addressed by both fields, but from different angles. Dissemination science examines how findings can be effectively communicated across diverse settings and populations, while IS investigates how interventions can be successfully adapted and integrated into various practice contexts. Rather than viewing these as separate domains, contemporary frameworks increasingly recognize their synergistic relationship - effective implementation often requires strategic dissemination, while successful dissemination must consider implementation barriers and facilitators [15, 16]. 

Another feature of IS is the clear distinction that is made between the practice innovation that is being promoted and the strategies that are being used to facilitate its adoption. In classic IS, the innovation itself is assumed to have already been proven effective (“evidence-based”) and not to be in need of further evidentiary proof. The emphasis, therefore, is on evaluating the effectiveness of the implementation strategies that can be individualized to different sites of care, or changed over time in response to evolving needs– which would never be contemplated in other sorts of empirical inquiry, such as a randomized trial. This is part of how IS emphasizes real-world generalizability, potentially at the cost of de-emphasizing internal validity. This is a particular point of contrast with randomized trials, which emphasize internal validity, often at the expense of generalizability.

Finally, IS tends to focus on more ambitious and complex problems, and may therefore require dedicated research funding, whereas simpler QI approaches may suffice for less complicated problems or less ambitious goals. The development of IS was intended to provide an approach to help address these more challenging issues, as well as to provide avenues for grant funding and publications for those undertaking such studies. The possibility of grant funding and publications, in turn, may help attract a different caliber of project leaders, namely those with research experience and academic careers. It is important to emphasize, however, that to be successful, IS efforts must involve close cooperation between IS experts, healthcare leaders, and front-line health workers. IS is therefore not merely a set of methodologies to be taught within the academy, but is meant to be used to address real-world problems in cooperation with key stakeholders.

IS relies on related fields for what has been called pre-implementation [17, 18]. In order to identify a quality gap, there must first be consensus regarding what would constitute high-quality care. Thus, we need evidence regarding efficacy and safety of various treatments, which usually comes from clinically-focused research [17, 18]. We also need evidence regarding how closely health systems are approaching this ideal, as opposed to continuing to deliver care that is not in line with current evidence. This, in turn, is the province of health services research [17, 18]. Thus, we need clinical research and health services research in order to even know where to begin, by identifying areas in need of improvement through IS. However, producing clinical evidence and health services research is not enough to address the challenges of promoting the uptake of existing evidence into routine clinical care. IS was created to fill this gap.

### Increasing reliance on implementation science in many nations

Health systems worldwide are increasingly adopting IS to enhance healthcare delivery and outcomes. The World Health Organization (WHO) and the Global Alliance for Chronic Diseases are leveraging implementation science to optimize care for non-communicable diseases [19]. Countries exemplifying strong IS efforts, in both training and research funding streams, include the United States, Denmark, Germany, the Netherlands, the UK, Switzerland, Canada and Australia [20]. Significant research funding, for example, has been made available in these countries by major organizations such as the National Institute for Health Research in the UK, the National Institutes of Health (NIH) in the USA, the Canadian Institutes of Health Research, and Innovationsfond in Germany [20]. These funds have supported the establishment of large research programs, such as the Collaborations for Leadership in Applied Health Research and Care (CLAHRCs), which established 13 centers across the UK between 2014 and 2019 to develop and conduct applied health and care research across the British National Health Service (NHS), and to translate research findings into improved outcomes for patients.

Additionally, professorships and training programs in implementation science and quality improvement have been developed, particularly in North America, but also in other regions. The National Institute of Health’s Office of Behavioral and Social Science Research, for example, created the Training Institute for Dissemination and Implementation Research in Health (TIDIRH) which was expanded to disease specific areas like cancer, as well as to other countries such as Ireland and Australia [21]. Finally, to expand the impact of IS, fullfill the United Nations Sustainable Development Goals (SDGs), and improve global health equity [22], the Lancet launched in 2023 its Comission on Evidence Implementation in Global Health. The comission’s aim is to increase the uptake of IS in low and middle-income countries, as IS is crucial for evidence based uptake and reducing health and health care inequities [22]. 

### Building capacity for implementation science: the example of the Veterans Health Administration in the United States

The Veterans Health Administration (VHA, also sometimes known as the VA) delivers healthcare to approximately 10 million veterans of the US armed services. In addition to healthcare delivery, VHA has long invested in data collection, health services research, and efforts to use these as a basis for ongoing evaluation and system improvement [23]. Despite these efforts, there was a desire on the part of VHA leaders to push improvement processes even further, beyond simple evaluation and into deliberately changing the system. Together with several early pioneers of the budding field of IS, the VHA decided to establish a dedicated research institute to support IS, with research grants and a commitment to developing investigator capacity over time [24]. The VHA established the Quality Enhancement Research Initiative (QUERI) in 1998 to support IS; the program has continued to grow since then and has been a scientific “home” and funding source for thousands of projects and hundreds of investigators [25], many of whom have in turn mentored other younger investigators. QUERI-affiliated investigators have helped VHA tackle its most pressing and complex concerns, including improved delivery of treatment for opioid misuse, suicide prevention, improved management of acute stroke, and combating obesity through increased physical activity [25]. 

One example of a tangible benefit of VHA’s investment in the QUERI program is the Stratification Tool for Opioid Risk Mitigation (STORM). STORM is a tool developed to help address a pressing problem of opioid-related morbidity and mortality in the VHA system (and the United States in general). The STORM investigators had developed a model to predict which patients receiving prescription opioids would be at the highest risk for overdose and suicide events [26]. In many research contexts, the development and publication of this model would be the end of the research trajectory. However, QUERI investigators then oversaw a three-year randomized study of the implementation of this tool, and were able to demonstrate through a sophisticated stepped-wedge study design [27] that STORM led to a decrease in all-cause mortality among those assigned to use it, compared to control patients [28]. They continued to the step of developing training, technical assistance, and academic detailing efforts to support widespread use of this tool as part of the national roll-out in the VHA [29]. Thus, the STORM research team took two important steps after the epidemiological research had concluded– a real-world demonstration of impact and tools to support a system-wide scale up. Had the VHA continued its research program in 1998 without investing in building the QUERI program, the course of this research would likely have ended with the prediction model.

### Building capacity for implementation science: the example of Intermountain Healthcare in the United States

Intermountain Healthcare (Intermountain) is an integrated health system serving diverse urban and rural communities primarily in the Intermountain West region of the United States, including Utah, Idaho, Nevada, Colorado, Montana, Wyoming, and Kansas. It operates 33 hospitals, including a virtual hospital, and 385 outpatient clinics. Since the early 1990s, the system’s Healthcare Delivery Institute (HDI) has been charged with promoting clinical best practices across the organization. Because of dissatisfaction with existing tools’ fit to the organization, and in an effort to increase the uptake and implementation of evidence-based practices, Intermountain’s HDI developed a 2-pronged approach that included an advanced training program and fellowship dedicated to IS [30], as well as a unique evidence based practice implementation model called the Clinical Best Practice Integration Model (cBPI) [31]. The cBPI model includes five steps of search/develop/identify, define and certify, measure and report, implement/drive high adherence and sustain. cBPI has been scaled across the organization and across different specialties. Clinical Best Practice Integration (cBPI) consultant teams, that include implementation scientists and system engineers, support clinical teams that identify relevant evidence-based practices [32]. Over the years, similar to the VA, Intermountain has used its IS infrastructure to improve and address its most pressing clinical challenges in serving remote populations such as acute stroke care, head trauma, and acute respitory care [33, 34]. 

A current example of how Intermountain’s IS infrastructure is driving the organization’s care delivery is adherence to stroke protocols. Over the past two decades, Intermountain worked to implement evidence-based stroke care across its system, with mixed success, especially in emergency departments at non-stroke centers. Using its cBPI model, Intermountain created a centrally coordinated system-wide implementation initiative in which each hospital worked with the central cBPI consultant team to assess what implementation strategies would work best for deployment of the protocol within their emergency department. The type III effectiveness-implementation stepped wedge design study found overall improvememnt in all participating centers, with the biggest improvement in in rural and frontier non-stroke centers. This study demonstrated how a centrally led but locally customized approach can be utilized to improve acute stroke care across the very diverse emergency departments in the Intermountain system [35]. 

### Has implementation science proven its value?

Those who are new to the ideas of IS often ask if the field has demonstrated that it has improved patient outcomes or produced economic benefits. The answer to this question is necessarily complex. One possible answer would be to say that the immediate goal of IS is to promote rapid and complete uptake of evidence-based practices. To the extent that these evidence-based practices have been shown to improve patient outcomes, and to the extent that IS helps increase the uptake of them, then Implementation Science has benefited patients.

However, there is another level on which this question could be asked: how can we know that the approaches of IS are indeed promoting uptake of evidence-based practices better than some other approach could be achieving? It would be hard to construct a counterfactual to answer such a question– since the influence of IS has grown considerably, it would be hard to find a health system that has been completely untouched by it, which could serve as a control.

There have been some notable efforts to assess the value and contribution of large-scale IS programs. For example, the VHA QUERI program, discussed above, was the topic of a large summative evaluation of its cumulative impact [36]. Similarly, the CLAHRC program in the UK, also discussed above, was the topic of a large summative evaluation of cumulative impact and lessons learned [37]. However, there is a need for more such evaluations. While those involved in IS may be convinced of its efficacy, there is a need to increase the number of evaluations that can demonstrate economic and health outcomes impacts from these efforts. While there will continue to be challenges to such evaluations, especially in terms of finding appropriate counterfactuals to support an impact evaluation, this will remain an important goal for IS as it matures as a field. Indeed, prominent leaders in IS have called for IS projects to put greater emphasis on evaluation of economic impact, even relatively early in the implementation process [38, 39]. 

### The current state of implementation science in Israel

There are several entities that can be said to “own” the process of innovating in healthcare in Israel today. This includes the Ministry of Health, the four health funds (“kupot cholim”), individual hospitals, institutions similar to hospitals such psychiatric or rehabilitation hospitals, and public health systems such as Tipat Chalav. All of these, at various times, have pursued various innovations in care delivery [40]. However, it is not clear how much experience any of these entities have with IS or with another closely related field, such as Program Evaluation.

For example, all four of the health funds have research centers that perform research, help with operational analyses, and also serve as a central developer of innovation. That is, a single unit within the health fund is in charge of advancing both research and innovation within the organization. The Ministry of Health, and many large hospitals, also have their own research centers. However, to our knowledge, none of these research groups employ investigators with experience in IS. While research groups based at health funds, hospitals, and the Ministry of Health do publish extensively, they do not publish evaluations of program impact, even for large and otherwise extremely ambitious projects. For example, Israel’s Ministry of Health has been pursuing a program called Efshari Bari (A Healthy Option) for over a decade [41]. To our knowledge, there have not been any publications about the effectiveness of this program.

It is worth noting that most health-related research expertise in Israel has historically been in the field of epidemiology. Epidemiology is classically dedicated to pursuing internal validity at the expense of external generalizability, an orientation which may be at odds with the goals and methods of IS. This orientation toward epidemiology in Israeli public health is reflected in the disciplinary background and interests of the majority of the *faculty* at Israeli schools of public health, medicine, and nursing, the large proportion of *research grants* that support epidemiological studies, and the majority of the *publications* using Israeli data. Epidemiology certainly has an important place in the field of public health, but by itself it is unlikely to produce health system change– especially since it often concerns itself, quite properly, with determinants of health which occur entirely outside the health system.

Leaders at Israeli institutions such as hospitals, health funds, and the Ministry of Health also reflect this emphasis on epidemiology, because many of them studied at Israeli academic institutions. This emphasis, in turn, is reflected in the research products of Israeli institutions. The main products have traditionally been interventional and observational studies in the mold of epidemiology– with a special emphasis on randomized trials. Randomized trials are an extremely important part of the research enterprise, but they are not by themselves sufficient to produce health system change. The production of evidence of efficacy must be followed by evidence of real-world effectiveness, and then by efforts to actually implement change in the real world and measure impact. Otherwise, the results of randomized trials will result in publications– possibly high-profile publications– but will not contribute to improved care for patients.

We, the three authors of this Perspective, are among relatively few practitioners of IS in Israel today, having received training in IS during our graduate and post-graduate work outside Israel. While we are involved in training others, to our knowledge we are the only Israeli researchers who have successfully led grant-funded projects in this area. As an illustration of our current activities, one of us (AJR) is currently leading an implementation and evaluation of the use of video-chat to facilitate decisions about involuntary admission in several psychiatric emergency departments in Israel. Another (SS) is leading the implementation and evaluation of a national initiative to reduce diabetes and obesity-related health and healthcare inequities. MEE has led the Israel arm of the Open Stewardship research project, which evaluates and optimizes audit and feedback interventions to improve antimicrobial prescribing practices across human and animal health settings in Canada and Israel, and examines how these intervention strategies can be effectively tailored and scaled across different healthcare contexts and systems [42–45]. These projects are being performed using the methods of IS, and would be difficult to complete successfully without our IS expertise.

Throughout its history, Israel has benefited from important ideas that were brought in from outside– often by new immigrants or by academics who went abroad for some of their graduate or post-graduate training [46]. The prominence of epidemiology in Israel, in fact, owes its existence to several early “founders” who brought these ideas from outside. We think that it is high time for the field of IS to “catch on” in Israel– for the long-term good of Israel’s health system. Our vision is that, twenty years hence, Israel’s health system will be enjoying the fruits of its investments in building IS capacity [47]– as the VHA and Intermountain Healthcare are doing today.

### What would be needed to invest in implementation science in Israel?

Just as with efforts to change the health system for the better, we do not have the luxury to wait for Israel’s research focus to rebalance itself naturally. The development of IS capacity in Israel needs to be nurtured in several ways, which must unfold simultaneously, will be mutually reinforcing, and will require *deliberate effort and priority-setting* [21]. 

First, schools of public health, medicine, and nursing in Israel should deliberately move away from an often near-exclusive emphasis on epidemiology to encompass IS and related fields in their new hires– including adjacent fields of inquiry such as health services research, program evaluation, and clinical epidemiology. This is a long-term goal which will take at least a decade to really make an impact on molding faculties of public health, medicine, and nursing to emphasize a more balanced approach. This would involve hiring faculty who partly or fully focus on IS or adjacent fields. There is a need for training at various levels– masters’ degrees, PhD’s, and postdoctoral fellowships. It will be important not to ignore masters’ students, since they will form a large portion of the public health workforce, such as in the Ministry of Health. It would be equally possible to offer specific degrees in IS, or simply to have it as a “track” within the program, alongside existing “tracks” such as epidemiology. Even students who do not choose to focus on IS during their degree should at least be exposed to it, to help them feel comfortable with it as a part of public health and to help them at least know when to consult with someone who focuses on IS when certain challenges are encountered.

Some of the products of this system of training in IS will be employed within academia, continuing the process of training others and serving as a resource for some of the real-world implementation projects. Other graduates will leave academia and go to work at the institutions we mentioned earlier– the Ministry of Health, the health funds, and various hospitals. They will bring with them this expertise and perspective that comes with someone who has trained in IS and related fields.

Our vision would be that some practitioners of IS may be home grown in Israel– our former trainees in some cases, but that some will be hired from elsewhere. This may include immigrants to Israel, or Israelis who have trained abroad and brought this knowledge back with them. The existence of more faculty working in IS will eventually take on a momentum of its own, as larger numbers of potential mentors and colleagues can provide a fuller experience of collegiality to support career growth, both within academia and outside it.

Second, Israeli research funders need to become more familiar with IS, and may need to establish dedicated funding streams to support it– as the QUERI program did starting in 1998. Researchers who are located outside the “sweet spot” of a funder may feel the need to “disguise” their research as something other than what it actually is– which may make it hard to put together a successful proposal. In particular, a large percentage of Israeli research funding is explicitly designated for “pure” science and has standing instructions to reject any proposals whose purpose is “practical”– regardless of their merit or rigor. This would on the face of it make it difficult to find funding for an IS project in Israel, and may put pressure on researchers to try to disguise their IS projects as something other than what they are. The answer may be for funders to expand their “sweet spot” and develop ways to distinguish more vs. less meritorious IS proposals rather than summarily rejecting all of them, and/or for new funders to arise that are dedicated to IS. In addition, funding structures that truly aim to promote IS as part of the research community need to explicitly encourage cross-disciplinary collaborations, and collaborations between clinical leaders and research leaders, in recognition of the central importance of such collaborations in the success of IS efforts.

It may take many years for funders to change their orientation. The NIH has been increasing its commitment to funding and facilitating IS for over a decade, and this effort continues to this day [48]. Thus, anyone expecting Israel’s main research funders to increase their orientation to and acceptance of IS should be realistic about how long it will take, and how much effort must be put into making such a change. Given the dependence of many Israeli researchers on local funding sources, we do not think Israel can depend on external funders to fund our IS efforts. Funders from outside Israel, such as European Union-funded research or the NIH, do sometimes explicitly request work that involves IS or a related discipline [48, 49], but these grants can be hard for Israelis to obtain, and in addition the trans-national nature of such grants inherently diminishes their focus on addressing the challenges particular to Israel’s health system.

Third, Israeli research publications, including the Israel Journal of Health Policy Research itself, need to increase their receptivity to reviewing and publishing IS-related projects. Like any research discipline, IS has its own standards for what constitutes rigorous and meritorious research, and reviewers may differ in their familiarity with IS or their ability to relate to it or to appreciate its importance. Reviewing a manuscript about an IS project requires a sensitivity to evaluating this work on its own terms and not expecting it to be something that it is not. In time, IS in Israel may also benefit from the development of new and dedicated forums for publication, but we think the scope of the Israel Journal of Health Policy Research can and should encompass deliberate efforts to improve health care delivery, and a rigorous evaluation of their impact.

Fourth, as mentioned above, there are numerous entities that can and do serve as homes for innovation in Israeli healthcare. We think it would be wise for some of these entities to invest in building specific capabilities for IS and related fields. While some hospitals may be too small to attempt this, larger hospitals may be able to do it. The kupot themselves seem like prime candidates to build their own IS capacity, as does the Ministry of Health. It is important to remember that IS should not merely be forced to compete directly with groups dedicated to epidemiology and related pursuits, because the goals, methods, and people may be different. It seems natural, given the structure of healthcare in Israel, that some IS expertise may remain concentrated at the universities, but that some may also be located within the health system itself. Having more employers who can potentially hire and provide on-the-job training to IS practitioners can only enhance the continued viability of the field of IS in Israel.

Fifth, and finally, increased availability of data in the Israeli context would go a long way toward being able to identify what aspects of the health system are most in need of improvement through IS. While a discussion of data access is not the central focus of this manuscript, it seems quite likely that a limited ability to examine quality gaps in Israel is contributing to a limited interest in addressing them. In Israel, each of the four health funds controls the use of its own data, and each of the hospitals its own data. There is no database that reliably combines these data into an all-population database, as is done in some other countries [50]. In Israel, data access for research can be costly, and is subject to approval by the database owner, meaning that projects with a potential to uncover quality gaps may not be performed. It is long past time for Israel’s Ministry of Health to coordinate access to healthcare data by reasonable request to researchers affiliated with universities and equivalent non-profit organizations. As happened in the VHA, a tradition of self-evaluation and self-reflection can grow over time into a tradition of deliberately pursuing system improvements. But without a means of examining the current state of affairs, we have little chance of seeing clearly what even needs to be improved.

Having made these five recommendations, we will also point out that there are many important strengths in the Israeli health system that could greatly enhance the chances of success for IS. Israel is an innovative country with a highly educated workforce, which has had more than its share of economic success despite being surrounded by enemies [51]. Israel’s health workers, research workforce, and grant funders clearly have the ability to support a successful IS effort, if they decide to do so. Indeed, the four health funds, and the relatively integrated health system, have long been potent catalysts of health innovation [52], and could easily turn their attention to building a successful effort to study and improve the Israeli healthcare system if the right conditions were present to encourage such a move.

## Conclusions

Implementation Science is a scientific field of inquiry that aims to build generalizable approaches to pursue system change and close quality gaps more quickly. Health systems that have invested in building IS capacity have increased their ability to address more complicated and challenging issues more effectively, although this investment may take years to pay off. Today, Israel is relatively poorly developed in terms of its capacity to support IS. We recommend deliberate investments in building a more robust infrastructure for IS in Israel over time, which will pay off in the future. These steps would include (1) rebalancing university faculties toward IS over time to grow the community of practitioners; (2) creating centers of IS work within the health funds, the Ministry of Health, and large hospitals; (3) creating funding sources that are dedicated to, or at least friendly to, IS projects; (4) providing forums for publication of the results of Israel-based IS projects; and (5) increasing transparency in the Israeli health system by improving data access for researchers located outside the health funds. By pursuing these steps over the next decade or two, it is our hope that Israel can nurture an IS enterprise here that we can be proud of, and that can help transform our health system for the better.

## Data Availability

Not applicable.

## Abbreviations

cBPI: Clinical Best Practice Integration
CLAHRCs: Collaborations for Leadership in Applied Health Research and Care
HDI: Healthcare Delivery Insitute of Intermountain Healthcare
IS: Implementation Science
NHS: National Health System of the United Kingdom
NIH: National Institutes of Health
PDSA: Plan-Do-Study-Act
QI: Quality Improvement
QUERI: Quality Enhancement Research Initiative
SDGs: Sustainable Development Goals of the World Health Organization
STORM: Stratification Tool for Opioid Risk Management
TIDRH: Training Institute for Dissemination and Implementation Research in Health
VHA: Veterans Health Administration
WHO: World Health Organization
